# Reconsidering the nature and mode of action of metabolite retrograde signals from the chloroplast

**DOI:** 10.3389/fpls.2012.00300

**Published:** 2013-01-04

**Authors:** Gonzalo M. Estavillo, Kai Xun Chan, Su Yin Phua, Barry J. Pogson

**Affiliations:** ARC Centre of Excellence in Plant Energy Biology, Research School of Biology, The Australian National UniversityCanberra, ACT, Australia

**Keywords:** retrograde signaling, metabolite, drought, high light, gene regulation, 3′-phosphoadenosine 5′-phosphate, methylerythritol cyclodiphosphate, β-cyclocitral

## Abstract

Plant organelles produce retrograde signals to alter nuclear gene expression in order to coordinate their biogenesis, maintain homeostasis, or optimize their performance under adverse conditions. Many signals of different chemical nature have been described in the past decades, including chlorophyll intermediates, reactive oxygen species (ROS), and adenosine derivatives. While the effects of retrograde signaling on gene expression are well understood, the initiation and transport of the signals and their mode of action have either not been resolved, or are a matter of speculation. Moreover, retrograde signaling should be considered as part of a broader cellular network, instead of as separate pathways, required to adjust to changing physiologically relevant conditions. Here we summarize current plastid retrograde signaling models in plants, with a focus on new signaling pathways, SAL1-PAP, methylerythritol cyclodiphosphate (MEcPP), and β-cyclocitral (β-CC), and outline missing links or future areas of research that we believe need to be addressed to have a better understanding of plant intracellular signaling networks.

## Introduction

Chloroplasts originated from free-living cyanobacteria that were engulfed by the eukaryotic cell ancestor (Delwiche, 1999). This endosymbiotic event resulted in the transfer of thousands of genes from the cyanobacterial plastid ancestor into the nuclear genome of the host (Goksoyr, 1967; Martin et al., 2002). As a result of this gene transfer, the stoichiometry of nuclear and chloroplastic encoded polypeptides functioning in the plastids requires the coordination of both genomes. One such regulatory mechanism, called anterograde signaling, entails the coordination of plastid gene expression (PGE) by nuclear encoded proteins (Woodson and Chory, 2008). The reverse mechanism, or retrograde signaling, requires the transfer of signals from the plastids to the nucleus to regulate the expression of genes encoding both plastid-localized (Strand et al., 2003) and other proteins involved in many cellular processes (op den Camp et al., 2003; Koussevitzky et al., 2007; Pesaresi et al., 2009).

The biogenesis and functioning of plant organelles are coordinated with nuclear gene expression. Early stages of chloroplast development require the establishment of “biogenic” signals to coordinate the production of photosynthetic complexes and membranes (Oelmuller et al., 1986; Pogson et al., 2008). On the other hand, “operational” signals are important for the normal functioning of chloroplasts in mature plants (Pogson et al., 2008). Mature chloroplasts can act as environmental sensors as adverse environmental conditions such as high light (HL) and drought can cause energy imbalances leading to oxidative stress that will impair organellar and cellular function (Wilson et al., 2006). As a result, nuclear and plastidic gene regulation is required to reach homeostasis.

The first evidence that chloroplasts can regulate nuclear gene expression was obtained in the *albostrians* barley mutants deficient in plastid ribosomes (Bradbeer et al., 1979) and in Brassica plants treated with spectinomycin, an inhibitor of organelle protein synthesis (Zubko and Day, 1998). In both cases, bleached leaves were produced with decreased amount of nuclear encoded chloroplast proteins. These observations lead to the proposal that perturbation in plastidic processes give rise to plastid products, or signals that can control cytosolic protein translation.

Since then, different types of retrograde signaling pathways, depending on the trigger sources and signals, have been reported. One signaling pathway is associated with tetrapyrrole biosynthesis intermediates, like Mg-ProtoporphyrinIX (Mg-ProtoIX) (Strand et al., 2003) and haem (Woodson et al., 2011). A second type is initiated by changes in redox potential at the electron transport chain (Fey et al., 2005; Pfannschmidt et al., 2009). The production of reactive oxygen species (ROS), such as hydrogen peroxide (H_2_O_2_) and singlet oxygen (^1^O^−^_2_) by excess oxidative power is a third mechanism that can trigger specific changes in nuclear gene expression (Apel and Hirt, 2004; Galvez-Valdivieso and Mullineaux, 2010; Suzuki et al., 2012). Finally, there is a type of retrograde signaling associated with PGE (Bradbeer et al., 1979; Nott et al., 2006).

The “classical,” or linear, model of retrograde signaling describes that specific signals produced in the organelles by different developmental and environmental cues are able to move into the nucleus where they elicit specific gene regulation. Although there is a good understanding of some of the triggers, the nature, and the final outcomes related to gene expression for some of these proposed retrograde signals, some of the signals are still debated or their mechanism of actions poorly understood. This *Perspectives* article presents a synopsis of the current knowledge of metabolite plant retrograde signals with a focus on the recent reports of novel signals. We also attempt to identify missing gaps in current models and provide suggestions for future directions of research. Readers are referenced to pertinent reviews for further details regarding other signaling pathways (Apel and Hirt, 2004; Pogson et al., 2008; Woodson and Chory, 2008; Galvez-Valdivieso and Mullineaux, 2010; Pfannschmidt, 2010; Barajas-López et al., 2012).

## Classical retrograde signals: chlorophyll precursors

Classical retrograde signals in plants generally involved artificially stressing the plant cells by treating the plants with the herbicide norflurazon (NF), which is an inhibitor of carotenoid biosynthesis that can perturb chloroplast development (Foudree et al., 2010). A mutant screen for altered expression of the nuclear genes encoding plastidic proteins during chloroplast development gene led to the discovery of the *GENOMES UNCOUPLED* (*GUN*) mutants (Susek et al., 1993). *gun* mutants are defective in the chloroplast-to-nucleus signal transduction that represses the expression of photosynthesis-associated nuclear genes (PhANG) genes such as *Light Harvesting Complex b (LHCB)* during perturbations of chloroplast development by NF.

At least two intermediates in the synthesis of photosynthetic pigments can act as plastidic signals to regulate nuclear gene expression. Treatment of wild type plants with NF not only inhibits the expression of the PhANG (Susek et al., 1993) but concomitantly induces 15-fold the levels of Mg-ProtoIX, the first committed precursor of chlorophyll. Genetic inhibition of Mg-ProtoIX production, such as in the *gun2* and *gun5* mutants (Mochizuki et al., 2001), which are defective in tetrapyrrole biosynthetic enzymes, results in misregulation of 70 out of 182 genes normally down-regulated in NF-treated wild type plants (Strand et al., 2003). Moreover, pharmacological approaches to accumulate Mg-ProtoIX, either by increasing its amount in the *gun2* and *gun5* mutants, or by feeding it to wild type plants, strongly support the hypothesis that Mg-ProtoIX is required for chloroplast-to-nucleus communication during early plant development (Strand et al., 2003; Kindgren et al., 2012).

Haem is a product of tetrapyrrole biosynthesis that acts as a positive retrograde signal from plastids in algae (von Gromoff et al., 2008). Evidence that haem could also be a potential signal in higher plants came from over expression of the *Ferrochelatase 1* (*FC*) gene in the gain-of-function *gun1-6D* mutant. FC1 over expression leads to the accumulation of PhANGs in the presence of NF (Woodson et al., 2011). This “*gun*” phenotype can be rescued pharmacologically by decreasing the FC activity with Fe^2+^ chelator dipyridyl (DP). This response seems to be specific to the activity of FC1, as over expression of the other chloroplast-localized FC2 did not increase PhANG expression. Unexpectedly, and unlike Mg-ProtoIX, a reduction in total haem, rather than accumulation, occurs in the FC1 OX, and wild type plants after NF treatment (Woodson et al., 2011). This finding is in agreement with the ineffectiveness of haem feeding in seedlings to silence *LHCB* (Strand et al., 2003), but is in contrast with the effect of hemin (a more stable Fe substitute), which promoted global changes in gene expression in *Chlamydomonas* (von Gromoff et al., 2008; Voss et al., 2011). It is proposed that FC1 acts on specific chloroplastic haem pool, which can act as a positive retrograde signal exported from the chloroplasts (Woodson et al., 2011). Although haem can be exported from isolated chloroplasts (Thomas and Weinstein, 1990), the actual transport mechanism is unknown. The fact that there is no correlation between free haem levels and the *gun* phenotype indicates that the signaling haem may be bound to specific targets (Espinas et al., 2012). A possible scenario is that haem interacts with cytosolic or nuclear factors, such as in yeast (Zhang and Hach, 1999) or with haem-binding proteins to regulate gene expression. However, more work is required to identify downstream targets of haem and their mode of action.

Although initial evidence indicate that Mg-ProtoIX accumulates under oxidative stress in the cytosol and represses PhANG expression (Strand et al., 2003; Ankele et al., 2007; Pontier et al., 2007), some findings suggest a lack of correlation between the metabolite levels and gene expression (Gadjieva et al., 2005; Mochizuki et al., 2008; Moulin et al., 2008; Kakizaki et al., 2009; Zhang et al., 2012). Interestingly, another recent finding shows that oxidative stress induced by NF can induce transient accumulation of tetrapyrroles with concomitant repression of *Lhcb* in adult plants (Zhang et al., 2011). The contradictory results could be explained by differences in experimental conditions or technical issues related to the quantification of tetrapyrroles.

Mg-ProtoIX accumulation specifically inhibits the expression of genes harboring the CUF1 (G-Box) *cis*-element (Strand et al., 2003). Two alternative models have been proposed whereby an increase of Mg-ProtoIX promotes either the release of a transcriptional activator or the binding of a repressor (Gray, 2003; Strand et al., 2003). While the potential for Mg-ProtoIX to move into the cytosol has been actively debated, recently more details of a possible mechanism of action mechanism have been described. Mg-ProtoIX was found to bind to the cytosolic heat shock 90-type protein (HSP90) and inhibit the ATPase activity of HSP90 *in vitro* (Kindgren et al., 2012). Feeding and genetic experiments confirmed that the *gun* phenotype triggered by oxidative stress is partially suppressed when HSP90 is silenced and that it requires the action of *Long Hypocotyl 5* (*HY5*) (Kindgren et al., 2012). Interestingly, HY5 binds to the promoter of photosynthetic genes (Lee et al., 2007). This type of signaling mechanism is analogous to that in yeast, where haem can interact with the complex HAP1-HSPs (Hon et al., 2001; Lee et al., 2002), which regulates proteins required for aerobic growth and oxidative damage control (Zhang and Hach, 1999). However, no direct interaction between HSP90 and HY5 has been reported yet in plants.

## Novel retrograde signaling pathways

**Figure 1:**
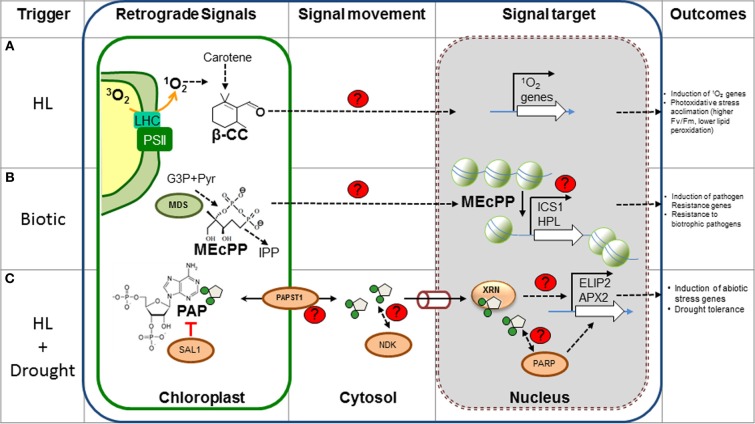
**New metabolic retrograde signaling pathways.** The different components of recently discovered plant retrograde signaling pathways are shown. **(A)** β-CC is most likely produced by oxidation of carotenes by ^1^O_2_ in the chloroplast and could diffuse through the membrane into the cytosol. Feeding β-CC results in the up-regulation of genes involved in stress responses, particularly those triggered by ^1^O_2_. It is speculated that the electrophilic carbonyl group could react with electron donors, such as sulfhydryl groups; however, the actual targets and mode of action are unknown. **(B)** MEcPP, an intermediary of isoprenoid precursors, is produced by MDS in the plastidic MEP pathway. MEcPP over accumulating mutants present high levels of SA and are resistant to biotrophic pathogens. MEcPP regulates the expression of the *HPL* and *ICS1* gene, with concomitant production of SA. Although the transport and action mechanism are unknown, MEcPP could promote chromatin reorganization and induction of transcription of target genes. **(C)** PAP levels are catabolically regulated by SAL1 in the chloroplast and PAP transport is probably mediated by PAPST1. Cytosolic PAP could diffuse to the nucleus via the pores (cylinder) where it inhibits nuclear *XRNs* and affects gene regulation of stress inducible genes (i.e., *APX2* and *ELIP2*). This mechanism is thought to play a role during drought, as PAP levels increase 30-fold. Other potential PAP targets (Nucleotide diphosphate kinase, NDK, and poly (ADP-ribose) polymerase, PARP, proteins) may mediate other aspects of signaling. The control mechanism of gene regulation by the XRNs proteins is a matter of investigation. Red lines, inhibition; black arrows, induction or activation; proteins involved in the signaling are indicated as ovals. Unknown components, processes or targets are indicated with red “?” or with dashed arrows. β-CC, β-cyclocitral; MEP, Methylerythritol phosphate pathway; MEcPP, methylerythritol cyclodiphosphate; MDS, MEcPP synthase; IPP, isopentenyl diphosphate; G3P, glyceraldehyde 3-phosphate; SA, salicylic acid; HL, high light. Figure adapted from Xiao et al. (2012) and Estavillo et al. (2011).

Despite a paucity of discovery of retrograde signals in the last decade, several signaling pathways have been recently proposed in the past 12 months. These novel signals include products of secondary metabolism (Estavillo et al., 2011; Xiao et al., 2012), oxidation products of carotenoids (Ramel et al., 2012), and dual localized proteins (Sun et al., 2011; Isemer et al., 2012) (Figure 1). These findings indicate that many metabolic pathways can act as potential sources of chloroplastic signals during different developmental stages of the plant or upon different stress responses. More importantly, unlike the previously discovered signals during artificial conditions, these new signals were identified during physiologically relevant stress responses like drought or HL. These new discoveries further support the concept that chloroplasts can indeed act as environmental sensors.

### The phosphonucleotide that acts as retrograde signal during high light and drought stress

The dinucleotide 3′-phosphoadenosine 5′-phosphate (PAP) is a novel metabolite discovered to play a role as a plastid signal during drought and HL stress in Arabidopsis (Estavillo et al., 2011). PAP accumulates in plants lacking the phosphatase SAL1/FRY1, which degrades PAP into adenosine monophosphate (AMP) and phosphate (Chen et al., 2011; Estavillo et al., 2011). PAP is also increased by up to 30 fold in wild type plants during drought (Estavillo et al., 2011) and exhibits a smaller increase in response to HL. The *sal1* mutants present up-regulation of 35% of the HL stress inducible genes, including *Ascorbate Peroxidase 2 (APX2)* and *Early Light Induced Protein 2 (ELIP2)*, altered metabolome and 50% increased survival under water limiting conditions (Rossel et al., 2006; Wilson et al., 2009). PAP is produced as a byproduct of sulfonation reactions catalysed by cytosolic sulfo transferases (SOTs), whereby sulphate is transferred from 3′-phosphoadenosine 5′-phosphosulfate (PAPS) to several metabolic substrates (Klein and Papenbrock, 2004). PAP levels are regulated by the dual localized SAL1 protein in both chloroplasts and mitochondria. Evidence that PAP can move between the chloroplast and nucleus was obtained by complementing PAP levels and *APX2* expression in *sal1* mutants with a SAL1 transgene targeted to the nucleus or chloroplast (Estavillo et al., 2011). Furthermore, a PAPS/PAP chloroplastic antiporter has now been reported that will facilitate exchange of PAP between the chloroplast and cytosol (Gigolashvili, 2012).

PAP most likely regulates gene expression by altering RNA metabolism mediated by 5–3′ exoribonucleases (XRNs). There are three XRNs in Arabidopsis; two encode the nuclear-localized XRN2 and XRN3 and are homologues of yeast Xrn2p/Rat1p. XRN2 and XRN3 act on uncapped RNAs like the excised hairpin loops in precursor miRNA transcripts (Kastenmayer and Green, 2000). The third gene, XRN4, encodes for a cytosolic XRN (homologous to yeast Xrn2p) that targets the resulting 3′ cleavage products of miRNA targets (Kastenmayer and Green, 2000; Souret et al., 2004). *SAL1* and nuclear *XRN* mutants share similar morphological phenotypes (Gy et al., 2007), have improved drought tolerance and more than 50% of altered genes are co-regulated (Estavillo et al., 2011). Additionally, PAP can inhibit yeast XRN activity (Dichtl et al., 1997; van Dijk et al., 2011) and transcriptional regulation by HL of *ELIP2* and *APX2* genes is similar in Arabidopsis *sal1* mutants (Estavillo et al., 2011). The mechanism by which XRNs modulate specific transcripts, such as *APX2* during stress (i.e., impact on RNA post-transcriptional regulation, “degradome,” etc.) is unknown and merits further research.

### Isoprenoid precursor mediates abiotic stress gene regulation

A genetic screening for the constitutive expression of the stress inducible nuclear gene encoding the plastid-localized hydroxyperoxidase lyase (HPL) identified the isoprenoid precursor methylerythritol cyclodiphosphate (MEcPP) as a potential signal from the chloroplast (Xiao et al., 2012). MEcPP is converted to hydroxymethylbutenyl diphosphate (HMBPP) by 1-hydroxy-2-methyl-2-(E)-butenyl-4-diphosphate synthase (HDS) during the production of universal isoprenoid precursors by the methylerithrytol phosphate (MEP) pathway in plastids (Rodríguez-Concepción, 2006). The mutant *ceh1* (*constitutively expressing HPL 1*) accumulates MEcPP and *HPL* transcript, with up-regulation of salicylic acid (SA) production, and that of the *ICS1* (encoding a SA-biosynthetic enzyme) transcript. This results in *ceh1* mutants being more resistant to infection by *Pseudomonas syringae*, a biotrophic pathogen. Targeted silencing of all the enzymes in the MEP pathway indicates that only the accumulation of MEcPP is responsible for SA accumulation and induction of *HPL*. Moreover, feeding experiments demonstrates that MEcPP can directly regulate *HPL*. Thus, it seems that unlike the mechanism mediated by chlorophyll precursors where the flux through the pathway is important for retrograde signaling (Woodson et al., 2011), the induction of *HPL* is specifically triggered by MEcPP, and not by other intermediaries of the MEP pathway. Finally, the accumulation of MEcPP is also induced by wounding and HL stress with concomitant up-regulation of the *HPL* gene.

Strong evidence supports the hypothesis that MEcPP is an operational signal, and like PAP, demonstrates that mature chloroplasts can respond to external perturbations under physiologically relevant conditions. The gene response to MEcPP is specific and it does not involve regulation of PhANGs (Xiao et al., 2012). Comparative global gene expression analysis between *ceh1* relative to wild type is needed to determine the extent of the signaling cascade by MEcPP and potential overlaps with other retrograde networks. Interestingly, oxidative stress in bacterial cultures induced MEcPP production (Ostrovsky et al., 1998) and MEcPP has been involved with the disruption of the interaction between chlamydial histone-like proteins and DNA. This would suggest that MEcPP could affect chromatin remodeling and gene expression (Grieshaber et al., 2006). However, direct evidence for MEcPP movement and its mechanism of action in plants is still lacking and deserves further investigation.

### A volatile retrograde signal

Carotenoids are considered to be one of the first lines of defense against ^1^O_2_ produced by the triplet excited chlorophyll during HL stress. Direct measurement of ^1^O_2_ and its regulated genes, indicates that production of ^1^O_2_ is one of the early responses to HL stress in Arabidopsis (González-Pérez et al., 2011; Ramel et al., 2012). Excess ^1^O_2_ under this condition leads to the production of oxidation products of carotenes. One of them, the volatile β-cyclocitral (β-CC), accumulates more than 1.5-fold after 1 h of stress treatment, which is accompanied by the induction of ^1^O_2_ stress-responsive genes (Ramel et al., 2012). More significantly, increasing doses of this volatile compound induces the up-regulation of ^1^O_2_, but not of H_2_O_2_, responsive genes. Microarray data also shows a striking specificity and similarity between gene expression profiles of β-CC plants and the *flu* mutant, which accumulates protochlorophyllide and presents a constitutive production of genes involved in oxidative stress by ^1^O_2_ (op den Camp et al., 2003). Finally, incubation of plants with β-CC prior to HL and cold stress prevented accumulation of lipid peroxidation and deterioration of PSII photochemical efficiency in a dose dependent manner, suggesting that β-CC increases photoxidative damage tolerance mechanisms. β-CC is a volatile short-chain compound, making diffusion a possible way of distribution from the place of origin to other parts of the cell. A likely action mechanism for β-CC is by reacting with sulfhydryl groups of proteins but more work is required to identify the target proteins and mode of action. Finally, it is possible that β-CC is a signal intermediate of the cascade triggered by ^1^O_2_ that relays the information to the cytosol.

### Mobile proteins

The signals described above could be considered as metabolite signals, which in some instances are capable of moving out from the organelle to regulate nuclear gene expression. However, two examples of chloroplast-localized transcription factors (TF) that translocate to the nucleus and alter gene expression under specific conditions have been recently described. Arguably, metabolite signals that could be modulated by specific proteins better fit in the “signal” category (Leister, 2012), but identification of the existence of the mobile TF involved in retrograde signaling confirms the complexity of the mechanisms required to regulate nuclear gene expression.

The chloroplast envelope-bound PTM (PHD type transcription factor with transmembrane domains) is involved in retrograde signal pathways that regulate PhANGs expression under different types of stress conditions (Sun et al., 2011). The plant homeodomain (PHD) specifically binds methylated histones (H3K4me3) to promote transcription of downstream genes (De La Paz Sanchez and Gutierrez, 2009). Under normal conditions, PTM resides in the outer membrane of the chloroplast. However, a shorter version of PTM protein lacking the transmembrane domain can be found in the nucleus after stress treatment with NF, lincomycin and HL. Stress conditions induce proteolytic cleavage of the full length protein and its translocation to the nucleus. Interestingly, the *ptm* mutant has reduced expression of *ABI4* and displays a *gun* phenotype (i.e., de-repression of *LHCB* in the presence of NF) similar to that of *gun1*, and *abi4* during HL treatment. The involvement of PTM in the same pathway as *GUN1* and *ABI4* was confirmed by analyses of the corresponding double mutants. Interestingly, the levels of H3K4m3 methylation in the *ABI4* promoter increased with the same stress treatments activating the PTM-dependent transcription of the *ABI4* gene. An unexpected observation was the lack of constitutive repression of *LHCB* in the absence of NF in plant expressing soluble PTM. Although this may indicate that additional GUN1 mediated signals may be required, this work demonstrates that PTM mediates several retrograde signaling pathways.

Another mobile protein that could potentially be involved in retrograde signaling is Whirly1 protein (Isemer et al., 2012). Arabidopsis Whirly proteins (AtWhy1 and 3) are required for plastid genome stability (Marechal et al., 2009) and the barley homologue interacts with intron-containing plastidic RNA (Melonek et al., 2010). The barley Whirly 1 (HvWhy1) protein is dual localized to both chloroplast and mitochondria (Grabowski et al., 2008) with the same molecular weight for both forms. Unlike PTM, the AtWhirly1 protein of the same molecular weight was found in both chloroplasts and nucleus of the same cell. It was recently shown that recombinant AtWhirly1 can move from chloroplasts to the nucleus by transformation of tobacco plastids with a HA-AtWhirly1 fusion protein (Isemer et al., 2012). This clever approach demonstrated that HA-AtWhirly1 protein can translocate from the plastid to the nucleus via an unknown mechanism. Although pathogenesis related genes are up-regulated in the transgenic lines (Isemer et al., 2012), suggesting the possible involvement of Whirly1 in retrograde signaling during pathogen attack, the movement mechanism of Whirly [i.e., via stromules, diffusion, transport (Krause et al., 2012)], and gene targets have to be further investigated.

## Dissecting signaling pathways

It is relatively easy to envision a linear type of mechanism whereby the available pool of signal interacts with target proteins to regulate gene expression (Estavillo et al., 2011; Kindgren et al., 2011; Xiao et al., 2012). The other option is that one signal can target many proteins or regulate the expression of many genes involved in different pathways. Rather than a linear succession of events, this would represent an intricate network which could provide more subtle levels of regulation under different and specific conditions (Leister, 2012). We outline some of the approaches utilized to discover new retrograde signaling pathways and components (Table 1).

**Table 1:** **Investigation of retrograde signaling pathways**.

**1. Signal sensing** ◦ Triggers for retrograde signals are not always clear ◦ What are the actual “stress sensors”?
**2. Signal movement** ◦ Confirmation of the movement: modulators targeted to specific compartments (Estavillo et al., 2011); genetically encoded biosensor; non-aqueous fractionation protocol coupled to MS. ◦ Identification of transporters (Gigolashvili, 2012) and study of their regulation in mutant plants or under different stress conditions.
**3. Signal targets and elucidating mechanisms of action**. ◦ Affinity chromatography coupled to proteomic analyses (Kindgren et al., 2011). ◦ Revertant screenings (Wagner et al., 2004; Šimková et al., 2012). ◦ Global changes in gene expression during fluctuating conditions (Brautigam et al., 2009; Voss et al., 2011). ◦ Investigation of epigenetic control during retrograde signal (Sun et al., 2011).
**4. Elucidating cross talk between signaling pathways** ◦ Comparison of global gene expression between different signaling mutants or under different triggering conditions (Schwarzländer et al., 2012) ◦ Study of epistasis (i.e., multiple mutants).
**5. Developing new systems for signal discovery**. ◦ Identification of new signals triggered by real physiological conditions or changing environments, like drought, light intensity and quality (Chan et al., 2010). ◦ Genetic screens

*Table indicating potential fields of research in retrograde signaling (1–5), and suggested approaches. Some examples are cited*.

### Triggering and movement of the signal

Unlike the case of photosynthetic derived signals, it is less clear how the different triggers are sensed in most retrograde signaling pathways. For example, how is the protease mediated cleavage of PTM regulated by HL (Sun et al., 2011), or the accumulation and transport of PAP induced by drought and HL (Estavillo et al., 2011)? These are difficult questions to address as they may involve the interaction of many different signaling networks.

The movement of the signal between organelles is a key feature of a metabolite, or protein capable of conveying information into the nucleus. Passive diffusion and active transport are the two most likely scenarios depending on the type of signal. However, monitoring this movement can be technically challenging. Analytical methods for metabolic profiling, such as cell fractionation in conjunction with non-aqueous fractionation, GC/MS- and LC/MS-based, and HPLC, or confocal microscopy for specific signals can be attempted. For instance, the observation that Mg-ProtoIX can accumulate in both chloroplast and cytosol suggests that this metabolite can be exported from site of origin, which may result in the repression of photosynthetic genes under stress (Ankele et al., 2007; Zhang et al., 2011). However, such approaches may be too harsh for labile signals or may not be possible due to cross-contamination of organelles (Estavillo et al., 2011; Woodson et al., 2011).

An alternative to direct measurements of the signal is the use of genetic approaches. Genetically encoded biosensors specific for putative signals, such as those developed for sugars and hormones (Frommer et al., 2009), could be used to measure the dynamics of the signal movement in different compartments. In a similar approach, the levels of the signal can be manipulated by targeting “sensors,” or “modulator” proteins to specific compartments and monitoring the changes in phenotypes. For example, targeting of a PAP degrading enzyme to the nucleus complements the molecular and morphological phenotype of *sal1* mutants, supporting the hypothesis that PAP moves in between organelles (Estavillo et al., 2011). Clearly, multiple lines of evidence are required to understand signal movement.

Identification of transporters that regulate the flux of the signal from the plastid into the cytosol is critical for understanding a signal transduction pathway. Although some of the proposed signals, like β-CC, could freely diffuse from the site of origin (Ramel et al., 2012), movement of others, like the highly charged PAP, or tetrapyrrole intermediates must necessitate specific transporters. There is good evidence that haem can leave intact chloroplasts (Thomas and Weinstein, 1990) and that Mg-ProtoIX can be found in the cytoplasm under stress conditions (Ankele et al., 2007). Although a couple of transport mechanisms have been proposed for Mg-ProtoIX (Moller et al., 2001; Larkin et al., 2003; Ankele et al., 2007), no chloroplast transporter has been described for haem. Very recently, co-expression analyses of genes related to glucosinolate metabolism lead to identification of PAPS transporter 1 (PAPST1), a transporter belonging to the mitochondrial carrier family that localizes to both the thylakoid, and plastid envelope (Gigolashvili, 2012). PAPST1 can transport ADP/ATP or PAPS /ATP, or PAP in an antiport manner *in vitro*. It still remains unclear whether this transporter plays any role in the PAP signaling pathway, especially during abiotic stress. Modulation of the transporter activity could be a major point of flux control, as also considered elsewhere (Leister, 2012).

### Hunting for signaling mechanisms and components

Affinity chromatography against putative signals coupled to proteomic analyses is a logical approach to dissect the components of the retrograde signaling pathways. This strategy rendered a large number of proteins associated to oxidative stress that bound to Mg-ProtoIX covalently linked gel matrix (Kindgren et al., 2011). This led to the proposal that a regulatory complex composed of HSP90 and Mg-ProtoIX could mediate gene expression (Kindgren et al., 2012). However, results can be misleading by non-specific interaction and proper controls and other additional lines of evidence are required (i.e., mutant analyses of potential targets, etc.).

Some of the retrograde mechanisms have been identified using inducible reporter genes, a common strategy for dissecting signaling pathways (Susek et al., 1993; Rossel et al., 2006; Estavillo et al., 2011). Another strategy to dissect new signaling components in order to understand mechanism of action is by performing reverse genetic screens of known signaling mutants (Šimková et al., 2012). However, the associated phenotypic screening can be misleading due to the potential pleiotropic effects of the signals. Additionally, study of co-regulated genes in a pathway can lead to the discovery of newer components (Gigolashvili, 2012).

### Cross-talk between pathways: linear or “one signal, many targets”?

Although the general view of retrograde metabolic signals is that of the “classical” model, where one signal acts through a rather linear pathway, it is likely that a “one signal, many targets” scenario is more common than anticipated, especially when considering metabolites that could bind to proteins. For example, 60% of genes misregulated in the SAL1 mutant *alx8* mutant do not seem to be affected by XRNs (Estavillo et al., 2011). This implicates that additional PAP targets exist in plants that may be important in controlling gene expression during stress. In fact, in plants and other organisms PAP can bind to other proteins, such as SOTs (Klaassen and Boles, 1997), nucleoside diphosphate kinase (Schneider et al., 1998) and poly(ADP-ribose) polymerase 1 (Toledano et al., 2012).

There are several recent examples of some classical retrograde signals converging with other networks such as light signaling (Ruckle and Larkin, 2009), plant immune signaling (Nomura et al., 2012), transition from cell proliferation to cell expansion (Andriankaja et al., 2012), cold acclimation (Crosatti et al., 2012), and ABA signaling (Koussevitzky et al., 2007) to name a few. It would be interesting to investigate whether the newer metabolic signals interact with other pathways (such as stomata regulation) that could be modulated by ABA, which levels increase upon HL, or drought stress.

## Concluding remarks

Several new metabolite retrograde signals have been recently proposed (Estavillo et al., 2011; Ramel et al., 2012; Xiao et al., 2012). New knowledge about the mechanism of other signaling pathways has also been gained (Fischer et al., 2012; Kindgren et al., 2012; Maruta et al., 2012; Šimková et al., 2012). Yet, none of the pathways are complete and for some transport or movement has yet to be demonstrated. Consequently, signal sensing and modulation is an important area of research. Additional efforts in analytical techniques for signal quantification and movement are critical for assessing the true “signal” nature of a metabolite. The fact that two new signaling pathways involve post-transcriptional regulation (Estavillo et al., 2011) and histone modifications (Sun et al., 2011) open an unexplored area for research: are there other cases of chloroplast-to-nucleus regulation where these types of gene regulation occur? The combination of deep sequencing technologies with the traditional reporter gene screen and multiple mutant approaches (including revertants) can give a more detailed picture of specific and overlapping networks for gene regulation. Finally, the development of new experimental conditions (i.e., controlled drought, finer manipulation of light quality and intensity) and technologies (i.e., biosensors, phenomics, revertant screening, and proteomics) will be instrumental in the discovery of new, true signaling components.

### Conflict of interest statement

The authors declare that the research was conducted in the absence of any commercial or financial relationships that could be construed as a potential conflict of interest.
